# Stromal Transcriptional Profiles Reveal Hierarchies of Anatomical Site, Serum Response and Disease and Identify Disease Specific Pathways

**DOI:** 10.1371/journal.pone.0120917

**Published:** 2015-03-25

**Authors:** Andrew Filer, Philipp Antczak, Greg N. Parsonage, Holly M. Legault, Margot O’Toole, Mark J. Pearson, Andrew M. Thomas, Dagmar Scheel-Toellner, Karim Raza, Christopher D. Buckley, Francesco Falciani

**Affiliations:** 1 Rheumatology Research Group, Centre for Muscoloskeletal Ageing Research, School of Immunity and Infection, College of Medical and Dental Sciences, University of Birmingham, Queen Elizabeth Hospital, Birmingham, B15 2WD, UK; 2 University Hospitals Birmingham NHS Foundation Trust, Mindelsohn Way, Birmingham, B15 2WB, UK; 3 Centre of Computational Biology and Modelling (CCBM), Institute of Integrative Biology, University of Liverpool, Crown Street, Liverpool, UK; 4 School of Cancer Sciences, College of Medical and Dental Sciences, The University of Birmingham, B15 2TT, UK; 5 Biological Technologies, Wyeth Research, Cambridge, Massachusetts 02140, USA; 6 MRC-ARUK Centre for Musculoskeletal Ageing Research, School of Immunity and Infection, College of Medical and Dental Sciences, University of Birmingham, Queen Elizabeth Hospital, Birmingham, B15 2WD, UK; 7 The Royal Orthopaedic Hospital NHS Foundation Trust, Birmingham, UK; 8 Sandwell and West Birmingham Hospitals NHS Trust, Dudley Road, Birmingham, B18 7QH, UK; Technische Universität Dresden, Medical Faculty, GERMANY

## Abstract

Synovial fibroblasts in persistent inflammatory arthritis have been suggested to have parallels with cancer growth and wound healing, both of which involve a stereotypical serum response programme. We tested the hypothesis that a serum response programme can be used to classify diseased tissues, and investigated the serum response programme in fibroblasts from multiple anatomical sites and two diseases. To test our hypothesis we utilized a bioinformatics approach to explore a publicly available microarray dataset including rheumatoid arthritis (RA), osteoarthritis (OA) and normal synovial tissue, then extended those findings in a new microarray dataset representing matched synovial, bone marrow and skin fibroblasts cultured from RA and OA patients undergoing arthroplasty. The classical fibroblast serum response programme discretely classified RA, OA and normal synovial tissues. Analysis of low and high serum treated fibroblast microarray data revealed a hierarchy of control, with anatomical site the most powerful classifier followed by response to serum and then disease. In contrast to skin and bone marrow fibroblasts, exposure of synovial fibroblasts to serum led to convergence of RA and OA expression profiles. Pathway analysis revealed three inter-linked gene networks characterising OA synovial fibroblasts: Cell remodelling through insulin-like growth factors, differentiation and angiogenesis through _3 integrin, and regulation of apoptosis through CD44. We have demonstrated that Fibroblast serum response signatures define disease at the tissue level, and that an OA specific, serum dependent repression of genes involved in cell adhesion, extracellular matrix remodelling and apoptosis is a critical discriminator between cultured OA and RA synovial fibroblasts.

## Introduction

Determining the mechanisms underlying persistence of disease in arthritis remains a significant challenge. The fibroblast-like synoviocyte (FLS) has become prominent as a mediator of disease persistence, supported by the persistent in vitro phenotype of these cells exemplified by the severe combined immunodeficiency (SCID) mouse model of cartilage invasion [1]. Osteoarthritis (OA) synovial fibroblasts have been investigated less extensively. Although OA FLS show some degree of hyperplasia [2, 3], they do not possess significant invasive properties. Transcriptomic analyses in conditions of high serum have revealed unique functional groupings of activated genes [4]. Such apparently imprinted characteristics are not unique amongst fibroblasts. Chang et al demonstrated retained fibroblast regional memory after in vitro culture [5]. However, this group also demonstrated a canonical serum response programme (CSR) in fibroblasts exposed to high (10% fetal calf serum (FCS)) compared to very low (0.1% FCS) serum [6, 7]. The presence of the CSR in publicly available gene expression profiles of cancer tissues robustly predicted metastasis and poor survival [7]. Further parallels exist between invasive tumours, wound healing and FLS. These include the expression of tumour associated markers such as Fibroblast activation protein (FAP) [8], Galectin-3 [9] and S100A4 (FSP-1) [10], evidence of distant spread of pathologically involved cells [11], and activation of pathways implicated in wound healing such as TGF*β* mediated myofibroblast differentiation as shown by Kasperkovitz et al [12]. The findings of Chang and Kasperkovitz [12] illustrate the potential of microarray analysis to generate hypotheses about the mechanisms underlying tissue microenvironments, but also highlight the critical effects of both anatomical site and serum exposure upon transcriptomic profiles. No study has attempted to examine diseased synovial fibroblasts in the context of serum stimulation. In order to fully understand the impact of serum in disease, we analysed transcriptional responses to serum of fibroblasts derived from three matched anatomical locations in populations of RA and OA patients. We identified a strict hierarchy of transcriptional regulation, and an OA synovium-specific signature, which is repressed in response to serum stimulation and represents important regulatory pathways involving control of extracellular matrix remodelling and apoptosis.

## Results

### A canonical fibroblast serum response signature discriminates between normal, RA and OA synovial tissues

RA FALS display an agressive phenotype, which involves increased invasiveness and resistance to apoptosis [13]. Because of this, the expansive synovial tissue called ‘pannus’ at the cartilage-bone interface has been suggested to share many hallmarks of cancer. However, there is no evidence of increased cell proliferation in RA compared to normal and OA FLS. Invasiveness, apoptosis and proliferation in RA FLS derived from patients share the same properties than their *in vivo* counterparts [1]. We therefore formulated the hypothesis that a fibroblast serum-response signature, linked to cancer clinical outcome, may be able to discriminate RA and OA derived synovial fibroblasts. Consistent with the current understanding of the biology of synovial FLS, we also hypothesize that migration and cell death but not proliferation may be the key difference between RA and OA derived FLS. We first tested this hypothesis by developing a statistical model predictive of disease type based on the original transcriptional signature developed by Chang et al [6]. We were able to map 95% of the genes originally described on a public domain dataset representing whole synovial tissue from normal, RA and OA individuals [14]. The hypothesis was indeed consistent with the prediction results as a K-nearest neighbour (KNN) based model was able to predict disease state with good accuracy. Normal samples were predicted with a sensitivity of 61% and a specificity of 95%. RA samples were predicted with a sensitivity of 88% and a specificity of 89% and OA samples were predicted with a sensitivity of 80% and a specificity of 81% (S1 Fig.). This observation therefore supported our hypothesis that fibroblast pathways involved in wound healing and neoplasia are relevant to the persistence of inflammation in RA and OA.

### A distinct hierarchy of anatomical site and serum response distinguishes fibroblasts derived from arthritis patients

**Fig 1:**
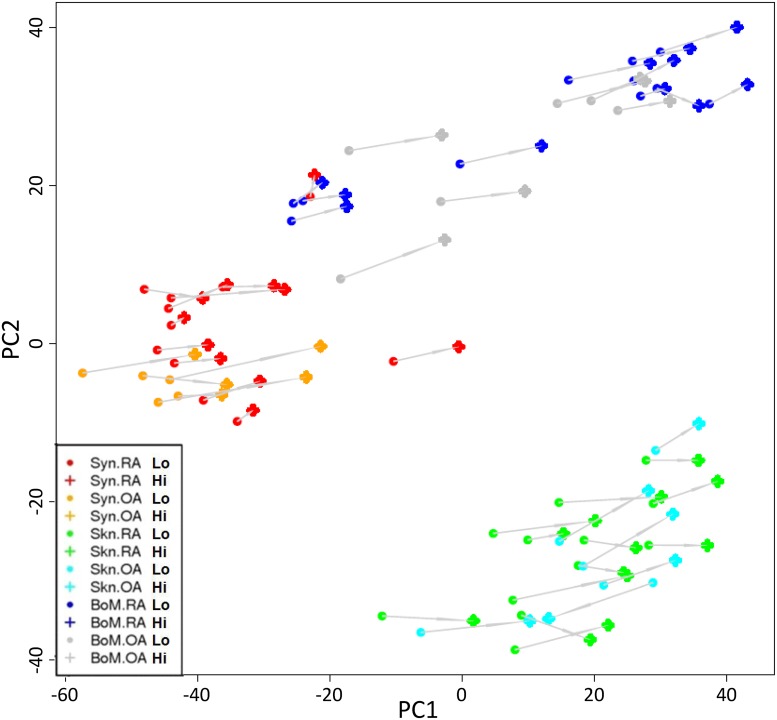
Identification of a hierarchy of molecular signatures in arthritis derived fibroblasts. The relative similarity of the different groups of fibroblast in a principal component (PC) plot is shown. Groups defined by disease state and anatomical location are indicated by coloured symbols whereas individual samples response to serum (Hi, High serum; Lo, low serum) is shown by a solid grey line. The PCA clearly separates synovium (Syn, red and orange) from bone marrow (BoM, blue and grey) and skin (Skn, green and light blue) on the first PC. Skin and bone marrow samples are only separated on the second PC. Additionally it can be observed that all samples are separated by their serum response mainly on the first PC.

Since the ‘core serum response’ signature (CSR) developed by Chang et al was based on fibroblasts derived from normal skin, lung and gum we reasoned that it may not fully represent the responses of synovial fibroblast populations in diseased joints. We therefore set out to experimentally define a serum response signature based on fibroblasts derived from both RA and OA patients. In order to take account of the documented effect of site on fibroblast transcriptional profiles and to address the possibility of multiple site involvement in systemic disease, we compared the serum response of fibroblasts of synovial origin to those of bone marrow and skin from the same individual. We set out to identify differentially expressed genes in at least one of the fibroblast groups. By using a SAM multiclass approach we could identify a signature of 3633 genes (FDR < 3% followed by a two-fold cut-off). Microarray gene expression was correlated with qPCR data for validation purposes (S2 Table). Principal component analysis (PCA) was used to visualise the relationship between the genes (Fig. 1). This unsupervised analysis showed that fibroblasts cluster primarily on the basis of anatomical location. Furthermore all matched low and high serum samples were separated on the first principal component (PC1, x-axis). By contrast, minimal clustering on the basis of disease was seen. These results provide the first evidence of a molecular hierarchy of transcriptional control, where site of origin is the dominant factor, followed by response to serum stimulation and disease status.

### Within anatomical locations, RA and OA fibroblasts are molecularly distinct and differ in their response to serum stimulation

While the PCA described above provides initial evidence for the existence of a molecular hierarchy of transcriptional control, it does not quantify the effect and it does not identify the specific genes linked to anatomical location, serum response and disease type. We therefore set out to directly quantify the differences in gene expression across all three levels of organisation and performed the following direct comparisons:

1. Anatomical location (Bone Marrow vs. Skin vs. Synovial tissue)
2. Serum Response (High vs. Low Serum)
3. Disease type (RA vs. OA)

**Fig 2:**
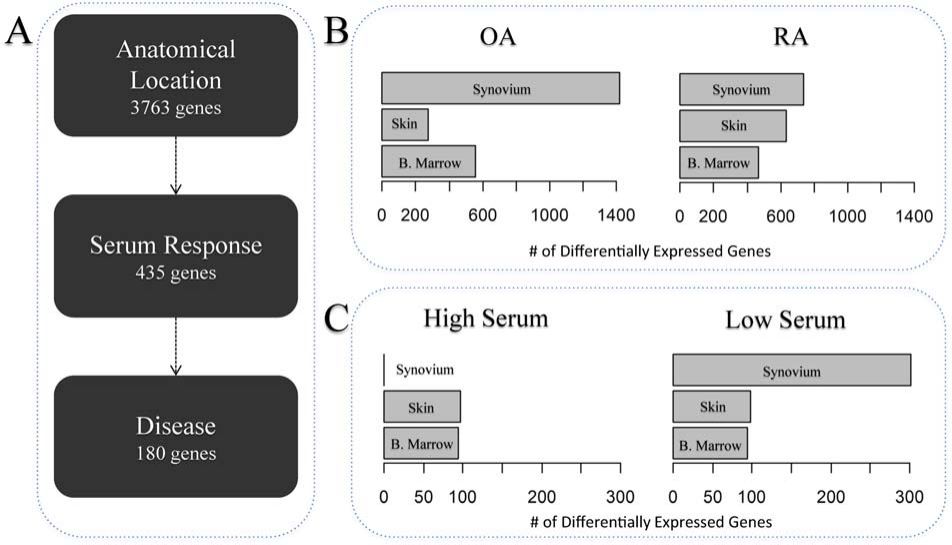
Differential gene expression analyses at tissue, serum and disease level. The figure shows the differential gene expression analysis performed within each level of the study. For each level of organisation we quantified the number of differentially expressed genes at an FDR < 5% followed by a 2-fold filter. This allowed us to devise a hierarchy of organisation that follows Anatomical Location then serum response and finally disease status (A). A more detailed tissue-by-tissue analysis to identify serum response genes in RA and OA fibroblasts (B) and to identify disease genes within high and low serum (C) was then performed. The arrows in panel A represent the direction of the hierarchy defined by the number of genes differentially expressed. BoM, Bone marrow.

**Fig 3:**
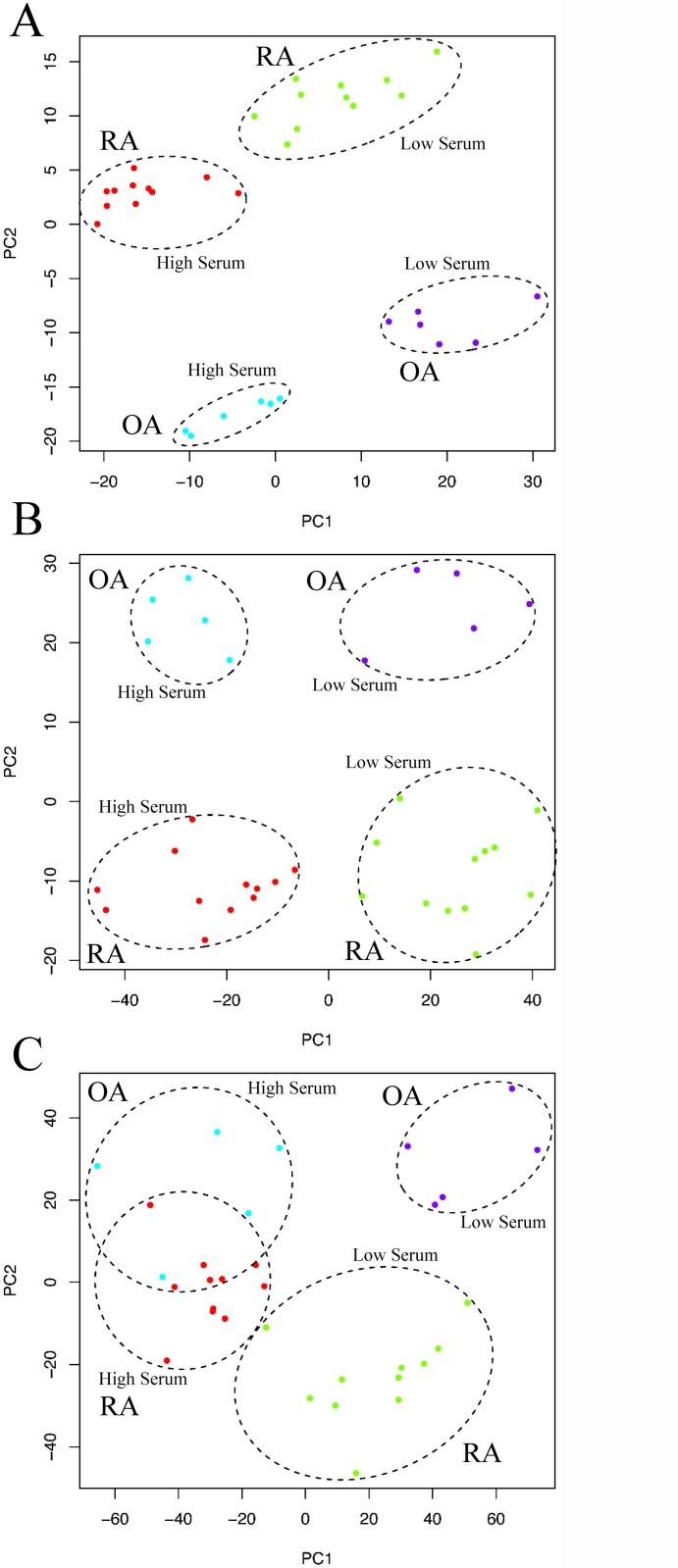
Serum response in arthritis derived fibroblasts within anatomical sites. The figure describes the relationships between RA and OA derived fibroblasts in low and high serum conditions using PCA. Bone marrow (A), skin (B) and synovium (C) derived fibroblasts are represented separately.

Fig. 2A summarized the results of this analysis showing that as expected anatomical location has the highest number of differentially expressed genes followed by serum response and finally disease (3763, 435 and 180 differentially expressed genes, respectively at < 5% and a 2-fold cut-off). We then focused within each anatomical location and performed a more detailed analysis. More precisely, within each tissue and within each disease group we identified genes differentially expressed between low and high serum (Fig. 2B). We found that in both RA and OA the synovial tissue has the highest number of differentially expressed genes (738 and 1419 genes respectively). Bone marrow tissues both expressed similar number of genes (RA: 469, OA: 558) while skin showed a much higher number of genes in RA (633) than OA (276) (Fig. 2B). We then identified within each anatomical location and within each serum level the number of differentially expressed genes between RA and OA samples (Fig. 2C). Here interestingly a large difference in the low serum but not the high serum condition could be observed in the synovial tissue (302 vs. 0 differentially expressed genes respectively). Skin as well as bone marrow both expressed similar numbers of differentially expressed genes (96±2) (Fig. 2C). The fold changes of differentially expressed genes were broadly similar between groups indicating that numbers of genes are a useful approximation of the degree of difference observed between transcriptional profiles. For each anatomical location we also performed a multi-class SAM analysis to identify genes that change in at least one of the different serum and disease types. We then visualised the resulting gene-lists using a PCA (Fig. 3). This clearly demonstrated that bone marrow and skin RA and OA fibroblasts responded to serum following parallel trajectories along the first principal component (PC) and were separated by disease predominantly according to the second PC (Figs. 3A and 3B), while RA and OA synovium fibroblasts separated along the second PC but converged in response to serum (Fig. 3C). In order to investigate the serum response overlap between bone marrow and skin fibroblasts, we extracted the most contributing genes within the first principle component, and found that 120 and 285 genes contributed to the first principle component of bone marrow and skin PCA analyses respectively, sharing 36 genes. This exclusive skin/bone marrow overlap suggested a specific synovial serum response.

### Serum response signatures defined by anatomical location and disease type define distinct functional profiles

**Fig 4:**
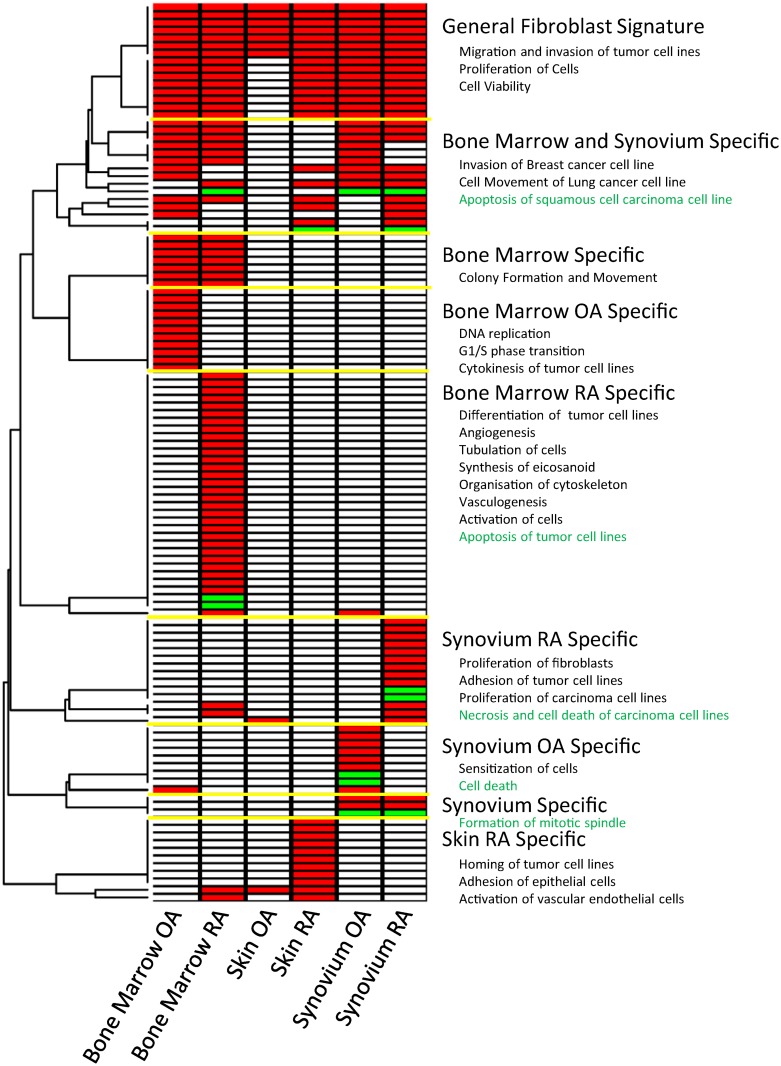
Functional classification of differentially expressed genes. Each set of differentially expressed genes in response to serum was submitted to Ingenuity and searched for functional terms with predicted increased (red) or decreased (green) activity.

Having defined the serum response signatures in each anatomical location and within each disease group we asked whether the serum response programs overlap significantly Samples derived from all three different anatomical locations of RA patients exhibited a greater overlap in serum response profile than OA samples, which exhibited more divergent, site-specific serum responses (11% vs 3% in common, supplementary S2 Fig.). Next, in order to address the biological significance of our findings we used the downstream effect analysis pipeline within the Ingenuity Pathway Analysis (IPA) knowledge management system. This algorithm assesses the probability that the observed transcriptional response is consistent with a phenotypic change. The rationale behind this approach is as follows: The IPA database contains experimentally observed causal relationships that provide a direction of effect between genes and functional terms. Given that generally a number of genes may be linked to any given function, a statistical approach to quantify this effect was developed by the IPA Team. Functional terms that have significantly more “increased” or “decreased” predictions are found by comparing the submitted gene lists with a random selection with equal probability. Fig. 4 represents a summary of the resulting functional terms linked to the serum response of anatomical locations and disease types. Most of the genes submitted by each comparison can be classified into functions relating to migration and invasion of tumor cell lines, proliferation of cells and cell viability (See S3 Table for more details). However there are a number functions specific to each anatomical site and disease type. Particularly bone marrow and synovial tissue show a number of overlapping functional terms such as invasion, synthesis of DNA and apoptosis. Moreover, bone marrow RA specific functions are one of the largest set of functions associated to a number of genes (423 genes associated to 30 increased and 104 genes to decreased functions) including differentiation, angiogenesis, vasculogenesis, activation of tumor cell lines and apoptosis of tumor cell lines (decreased). Within the synovial tissue, RA samples are characterized by proliferation of fibroblasts, adhesion of tumor cell lines and necrosis and cell death (decreased 145 genes in 2 functions). Similarly to RA, OA also regulate genes associated with apoptosis but with a much larger number of genes (246 genes associated to 2 functions). Interestingly, OA synovial samples are the only sample type with functions relating to sensitization of cells which may have an impact on their ability to respond to serum.

### Analysis of the synovium fibroblast response to serum identifies OA-specific repression of a tissue remodelling transcriptional signature

**Fig 5:**
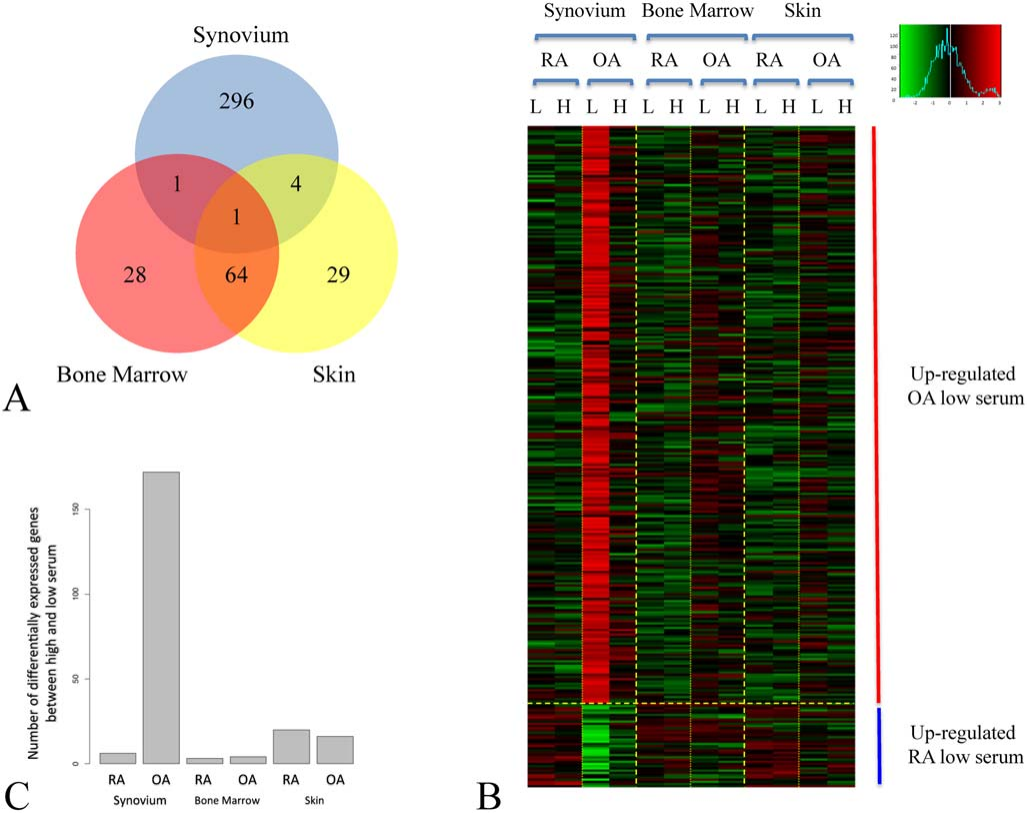
A synovium specific gene signature in low serum. Panel A shows a Venn diagram showing the overlap between genes differentially expressed in synovium, bone marrow and skin derived fibroblasts between RA and OA groups in the low serum state. The high degree of overlap between skin and bone marrow and the very different signature in the synovium are evident. Panel B shows the result of a two factor cluster analysis of the 296 synovium specific genes identified in Panel A. Panel C represents the number of statistically differentially expressed genes between low and high serum states within each tissue and disease. As expected OA synovium is associated with the largest number of differentially expressed genes.

**Fig 6:**
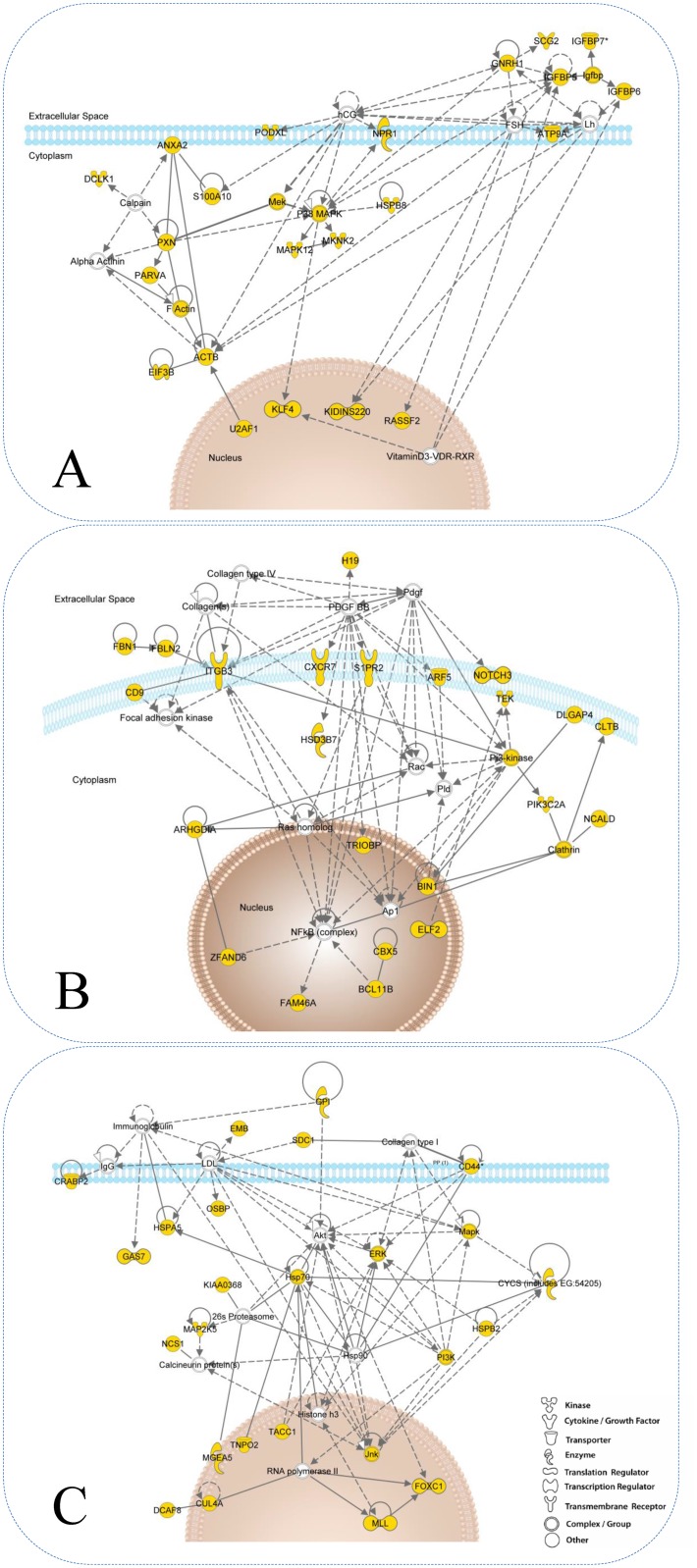
Ingenuity Network analyses. The three major networks identified were: (A) Actin cytoskeleton remodelling by extracellular insulin-like growth factor binding proteins through gonadotropic hormones. (B) ITGB3 signalling connected to differentiation and angiogenesis. (C) Regulation of apoptosis through CD44. Yellow nodes represent genes resulting from input whereas empty nodes represent added genes by Ingenuity. Dashed and solid lines represent indirect and direct relationships respectively.

A key question in arhritis research is whether RA and OA fibroblasts are different in their ability to respond to grow-factor signals. As shown in Fig. 3C, the transcriptional state of RA and OA derived fibroblasts converged in high serum culture conditions. We therefore set out to further characterize the difference in response between RA and OA in low serum conditions within the synovium and to assess whether these changes in expression follow serum stimulation. In the low serum condition, 302 genes were differentially expressed between synovial OA and RA fibroblasts. Genes significantly down-regulated in OA synovial fibroblasts during low to high serum transition and not included in classical GO classifiers included tumour suppressor and pro-apoptotic genes PTEN, SLIT3, TRERF1 (TReP-132), SHOX, SH3D19, ubr5 and EDD; genes with roles in cytoskeletal rearrangement supervillin, paxillin, and genes involved in ubiquitination and signalling included PIAS1, crebbp and the IGFBP family of genes. Genes regulated at higher levels in RA than OA fibroblasts included Secretogranin-II and tensin 3. Of the 302 genes, 98% were not differentially expressed in bone marrow or skin fibroblasts, confirming a synovium-specific response (Fig. 5A). Fig. 5B shows the relative expression in low and high serum of differentially expressed genes in synovial fibroblasts. The majority of genes (88%) were down-regulated in response to high serum in OA synovial fibroblasts. This relationship was also seen to a lesser extent in skin and bone marrow OA fibroblasts, while RA fibroblasts showed no convincing regulation of this gene list in response to serum. To define the biological relevance of the serum mediated synovium-specific disease signature, we performed a network analysis using Ingenuity Pathway Analysis software. This identified three inter-linked gene networks representing the control of actin remodelling through insulin-like growth factor associated proteins (IGFBP5, IGFBP6, IGFBP7 and GnRH1) (Fig. 6A), control of tissue remodelling by *β*3 integrin (Fig. 6B), and regulation of apoptosis through CD44 (Fig. 6C), each representing facets of the fibroblast phenotype in OA. This last observation shows that molecular signatures characterizing RA fibroblast serum response not only discriminate between RA and OA FLS but that the functional profile is related primarily to adhesion and migration pathways. This is consistent with our initial hypothesis.

## Discussion

We hypothesised that the CSR of Chang et al would differentiate persistent inflammatory RA from OA and normal synovium, and showed this to be the case at the tissue level. This observation places inflammatory arthritis at the centre of the long-standing debate regarding the relationship between wound healing, cancer and persistent inflammation [15]. Comparison of serum responses across site and disease led to the discovery of a hierarchy of transcriptomic signatures. The primacy of site as the strongest determinant of transcriptional fibroblast response fits with both Chang et al’s findings and clinical observations of organ specific disease [16]. Within anatomical sites, skin and bone marrow fibroblasts exhibited parallel serum responses regardless of disease. However the serum responses of RA fibroblasts exhibited greater overlap than OA fibroblasts and were broadly functionally aligned. This alignment of serum responses in RA could result from exposure to a systemic cytokine milieu, or from a field effect in mesenchymal precursors residing in bone marrow or local niches. Intriguingly, cultured skin and bone marrow fibroblasts also retained a robust transcriptional memory of disease following in vitro culture analogous to the disease memory exhibited by RA fibroblasts in the SCID mouse invasiveness model [1]. This is highly suggestive of underlying epigenetic mechanisms, just as site specific gene expression results from noncoding RNA (HOTAIR—a 2.2kb ncRNA fragment in the HOXC locus) driven epigenetic regulation of developmentally expressed genes [17]. We have previously shown that transcriptional profiles in lymphoid and peripheral fibroblasts may be modified under the influence of cytokine treatment [16]. The phenotype of all fibroblasts in RA may therefore result from in vivo modification of the serum response programme under the influence of persistent cytokine exposure. This finding has wider implications, not least for mesenchymal stem cell therapy, where allogeneic rather than autologous therapy may be more effective. Small numbers of genes linking fibroblast behaviour in RA and cancer have been previously identified [18]; our novel approach identified links to additional genes that are relatively silenced in RA compared to OA fibroblasts. An example is the tumour suppressor PTEN, the low expression of which at sites of synovial invasion and growth has been described by Pap et al. [19] SLIT3 specifically, along with other SLIT/ROBO family members, is inactivated by promoter hypermethylation in multiple human cancers [20], and antagonizes invasiveness of RA synovial fibroblasts [21]. SH3D19 codes for the tumour suppressor EBP that inhibits ras driven transformation in acute myeloid leukaemia [22]. ubr5 (EDD), a ubiquitin ligase, similarly acts as a DNA damage checkpoint with under-expression leading to cell cycle activation [23]. Responsiveness of these pathways in OA may help to explain the different clinical and radiological phenotypes seen in OA and invasive RA. Ingenuity pathway analysis identified three inter-linked gene networks: The first network represents the insulin-like growth factor signalling network. Transcription of the IGFBP family of genes which mediate profibrotic and migratory changes [24] has been described in a “low inflammation” group of RA fibroblasts by Kasperkovitz et al [12] and by Seki et al using differential subtraction techniques [25]. The second network represents the interaction between *β*3 integrin (ITGB3) and matrix components with signalling to differentiation and angiogenesis pathways. ITGB3 binds to multiple extracellular matrix components and growth factors including platelet-derived growth factor (PDGF), Collagens and Fibulin 2 [26–29]. Treatment of cells with PDGF-BB has been shown to increase the expression of ITGB3 [30] as well as increase recruitment of ITGB3 to the cell surface [28]. ITGB3:PTPN11 complexes also inhibit dephosphorylation of insulin-like growth factor-I receptor (IGF-IR), leading to inhibition of apoptosis via Akt and BclXL; hence decreased ITGB3 expression promotes apoptosis [31]. Angiogenesis related genes were consistently identified in RA fibroblast serum responses. However, although identified as a potential target in RA, an *α*v*β*3 monoclonal antibody (Vitaxin) was withdrawn after trials failed to show therapeutic benefit [32]. Furthermore, *β*3 and *α*v*β*3 integrin knockout mice exhibit enhanced pathological angiogenesis of implanted tumours while ITGB3 over-expressing models demonstrate small new vessels deficient in pericytes [33, 34]. The role of *α*v*β*3 integrins in angiogenesis therefore remains controversial. The CD44 pathway covers a broad spectrum of genes, including the MAPKinases and PI3Kinase that are all implicated in control of apoptosis and survival [35, 36]. Furthermore FOXC1 and CYCS are regulators of apoptosis, highlighted by gene ontology analysis as the most enriched term in this pathway. Our investigations shed light on the findings of Del Rey et al [4] who compared transcriptional profiles of synovial fibroblasts in high serum. They were surprised at the small number of differentially expressed genes seen, an explanation for which is provided by the serum conditions in which their experiments were conducted. Intriguingly, both studies identified unique aspects of the osteoarthritis synovial fibroblast phenotype, including cell adhesion and motility factors, and a conspicuous absence of proinflammatory cytokine pathways. We have demonstrated that serum response programmes segregate both tissues and fibroblasts from different sites and diseases. This may provide a molecular explanation for the ability of TGF*β* linked pathways, that form a part of this response, to sub-classify disease. These data also demonstrate at a transcriptome level that low serum conditions will increase the likelihood of identifying disease-specific signatures.

## Materials and Methods

### Analysis of synovial tissue datasets from public repository

We retrieved a dataset from the NCBI GEO database which contained synovial tissue biopsy samples from RA, OA and normal patients (http://www.ncbi.nlm.nih.gov/geo/query/acc.cgi?acc=GSE12021) [14]. The dataset was normalized using robust multi-array average (RMA) methodology; genes with expression close to the detection level were removed. We mapped cDNA common serum response (CSR) genes to Affymetrix® Ids using the ID converter facility in DAVID [37, 38]. Expression of successfully mapped genes was then determined in the GSE12021 dataset. To determine the ability of the CSR to classify synovial tissue from different diseases, tissue datasets were classified by CSR genes using a k-nearest neighbour (KNN) classification algorithm, with validation by a leave one out cross-validation (LOOCV) procedure.

### Patients and fibroblast culture

Fibroblasts were isolated as previously described [39] from synovium, bone marrow and skin tissue samples taken at the time of knee or hip replacement surgery from 12 rheumatoid arthritis patients meeting the 1987 ACR criteria [40] and 6 osteoarthritis patients diagnosed on the basis of characteristic x-ray findings and the absence of features suggestive of inflammatory arthritis (S1 Table). Only one hip sample was present in either disease group. Fibroblasts were maintained in fibroblast medium (consisting of 81.3% RPMI 1640, 10% FCS, 0.81x MEM non-essential amino acids, 0.81 mM sodium orthopyruvate, 1.62 mM glutamine, 810 U/ml penicillin and 81 μg/mL streptomycin) at 37°C in a humidified 5% CO2 atmosphere. Ethical approval for the use of this material was given by the ‘NRES Committee West Midlands—The Black Country’ and all patients gave written informed consent (LREC reference 5735). RA disease activity was quantified using the DAS28 disease activity score [41].

### RNA extraction and microarray hybridization

Cells were seeded in duplicate at the required density using 10% FCS. Once attached, the medium was removed and the cells washed in serum free medium before replacing with fibroblast medium, which was identical but contained either 10% or 0.1% FCS [6]. mRNA was extracted from all fibroblasts at passage 5, under identical conditions. In order to remove confluence of cells as a confounding factor, proliferation rates were measured, and appropriate numbers of cells seeded to achieve 75% confluent cultures at harvest. Fibroblasts were retrieved from culture by trypsin digestion and washed in fibroblast medium then PBS before resuspending in 200 μl of RLT RNA protection buffer (Quiagen). 102 fibroblast samples in all were prepared according to Affymetrix® manufacturer instructions using 50 ng total RNA, which was amplified using Nugen™ Ovation™ Biotin RNA Amplification prior to hybridization with U133 plus 2.0 Affymetrix® Genechips. All chips passed QC criteria and visual inspection of Expressionist thumbnail images. The data is available under GEO accession number GSE56409.

### Array validation using quantitative PCR

Synovial fibroblasts from 3 OA and 3 RA patients were cultured as above. Cells were trypsinised and RNA extraction performed using an RNeasy kit (Qiagen). RNA from 1×10^5^ fibroblasts was reverse transcribed using Superscript Vilo (Life Technologies). 1 μl of the resulting cDNA was added to the real-time PCR master mix (Applied Biosystems) and TaqMan primers/probes were used to measure the relative quantities of SLIT3, PIAS1 and TNS3 (VIC labelled) with GAPDH (FAM labelled) as control using an ABI 7900HT machine (Applied Biosystems).

### Dataset and data processing

Out of the 108 samples retrieved six samples did not meet quality control and hence did not undergo further analysis. These included 1x Bone Marrow RA low serum, 1x Bone Marrow RA high serum, 1x Skin OA low serum, 1x Synovial OA low serum, 1x Synovial OA high serum and 1x Skin OA high serum sample. Gene expression data from the Affymetrix® arrays were processed and normalized using RMA methodology. Data were processed to remove genes with expression close to the detection level.

### Identification of differentially expressed genes

Statistical analysis for microarrays (SAM) [42] as implemented by the R package (samr) was used to identify differentially expressed genes between groups. A cut-off of FDR < 3% and an additional cut-off of two fold change was applied on all differentially expressed gene sets. Initially we identified differentially expressed genes for each of the three levels of organisation (anatomical location, serum response and disease type). Here all samples belong to the given level were compared in either a “Multiclass” comparison (anatomical location) or a “Two class unpaired” comparison (serum response and disease type) using the SAM algorithm. The number of differentially expressed genes were then filtered to the previously mentioned thresholds. To identify genes differentially expressed in each tissue and within serum and disease types, we first subset the data into the three tissues. Then for each serum level we identify differentially expressed genes between RA and OA. Similarly for each disease type we identify differentially expressed genes between high and low serum. Apparent discrepancies in total gene numbers in tables compared to the text result from the common phenomenon of genes with multiple Affymetrix® IDs (See S1 Dataset for more details).

### K-nearest neighbour classification

To test whether the CSR signature is able to distinguish between normal, RA and OA samples retrieved from the public domain we applied a K-nearest neighbour algorithm as implemented by the knn.cv function in the ‘class’ library in the statistical environment R. This methodology uses a leave-one-out-crossvalidation. For each sample in the training set the k nearest neighbours are identified and the classification decided by majority vote. We have tested 2 < k < 10 and observed that the accuracy was independent of the value of k (standard deviation of the prediction accuracy over the values of k was < 0.05% in all comparisons).

### Data reduction techniques

Genes differentially expressed between all sample groups were visualised using Principal component analysis (PCA), a technique designed to represent highly multi-dimensional objects in a lower dimensionality space (Fig. 2). The first principal component (PC) on the x-axis represents the majority of the variance information, whilst the second PC on the y-axis, is orthogonal to the first PC and holds less variance. All data were mean centred prior to application of PCA using the prcomp function in the statistical environment R [43].

### Ingenuity Pathway Analysis (IPA) of differentially expressed gene sets

Gene sets derived from differential gene expression and Venn diagram analyses were analysed using the IPA application (Palo Alto, http://www.ingenuity.com) that uses the Ingenuity Pathways Knowledge Base (IPKB) to explore biological interaction networks. Each gene identifier is mapped to a global molecular network within IPKB. An algorithm then generates networks based on the connectivity of focus genes, limited to a maximum of 35 genes for simpler visualisation. For the downstream effect analysis gene lists and calculated fold changes were uploaded to the IPKB. For each biological function within the IPKB an activation z-score is calculated by the software. Here the molecular network within IPKB is interrogated and experimentally observed causal relationships between genes and function extracted. These connections provide a literature-derived direction of effect which is used in combination with the foldchange to calculate the activation state of the given functional terms. As multiple genes may be connected to a function a statistical approach is necessary to calculate its significancy as compared to a randomly derived variable. This variable is obtained by randomly choosing 1 or -1 with equal probability representing an “increased” or “decreased” state respectively. For each number of genes affecting the functional term the number of “increased” (*N*_+_) and “decreased” (*N*_−_) predictions is summed up for the random. This however assumes that there is only 1 possible downstream effect to a given function. To correct for this the IPAKB uses a weighted z-score statistics in which:

$$\begin{array}{c} w_{i}=\frac{|M_{activating}-M_{inhibiting}|}{M_{activating}+M_{inhibiting}+1} \end{array}$$ (1)

where *M*_*activating*_ and *M*_*inhibiting*_ represent the number of findings underlying an edges “activation” or “inhibition” respectively. This is then used to correct for the direction of change within the z score algorithm. The z-score can therefore be calculated as:

$$\begin{array}{c} z=\frac{x}{\sigma_{x}}=\frac{\sum_{i}w_{i}x_{i}}{\sqrt{\sum_{i}w_{i}^{2}}} \end{array}$$ (2)

## Supporting Information

S1 Fig Classification of normal and arthritis synovium biospies using a canonical serum response signature. The figure shows the probability of a synovium biopsy sample to be correctly classified as normal, RA or OA on the basis of a canonical serum response signature. The x axis represents each individual sample biopsy in the 3 groups and the y axis the probability of correct classification estimated using a leave one out cross-validation (LOOCV) procedure. The percentage of correct classification using a majority rule is indicated below the sample labels.
(TIFF)

S2 Fig Venn diagram representing disease specific serum response. A) Shows the Venn diagram for the response to serum in all tissues in OA. B) represents the serum response of all tissues in RA patients.
(TIFF)

S1 Table Patient demographic and disease data. RhF, rheumatoid factor; DMARD, Disease-modifying antirheumatic drug; MTX, methotrexate, weekly dose; D-PEN, D-penicillamine; Gold, intramuscular gold, monthly dose; AZA, azathioprine; SSA, sulphasalazine.
(DOCX)

S2 Table Fold changes in gene expression in OA versus RA synovial fibroblasts in low serum. Data were obtained from 3 synovial fibroblast lines. qPCR, quantitative PCR; PIAS1, protein inhibitor of activated STAT-1 (signal transducer and activator of transcription-1).
(DOCX)

S3 Table Functional associations in serum response. Overview of the Functional associations in the serum response between RA and OA fibroblasts within each anatomical site. The numbers represent “number of genes increased”/“number of genes decreased” within each term.
(DOCX)

S1 Dataset Results from the differential gene expression analysis. An excel file containing all results from the SAM analysis for all comparisons performed, including up and down regulated genes with their associated Gene ID, Fold Change and q-value (%).
(XLSX)
