# Supplementary figures and images for: Stromal Transcriptional Profiles Reveal Hierarchies of Anatomical Site, Serum Response and Disease and Identify Disease Specific Pathways
Source: PLoS One. 2015 Mar 25;10(3):e0120917. doi: 10.1371/journal.pone.0120917 (PMC4373951; doi:10.1371/journal.pone.0120917)

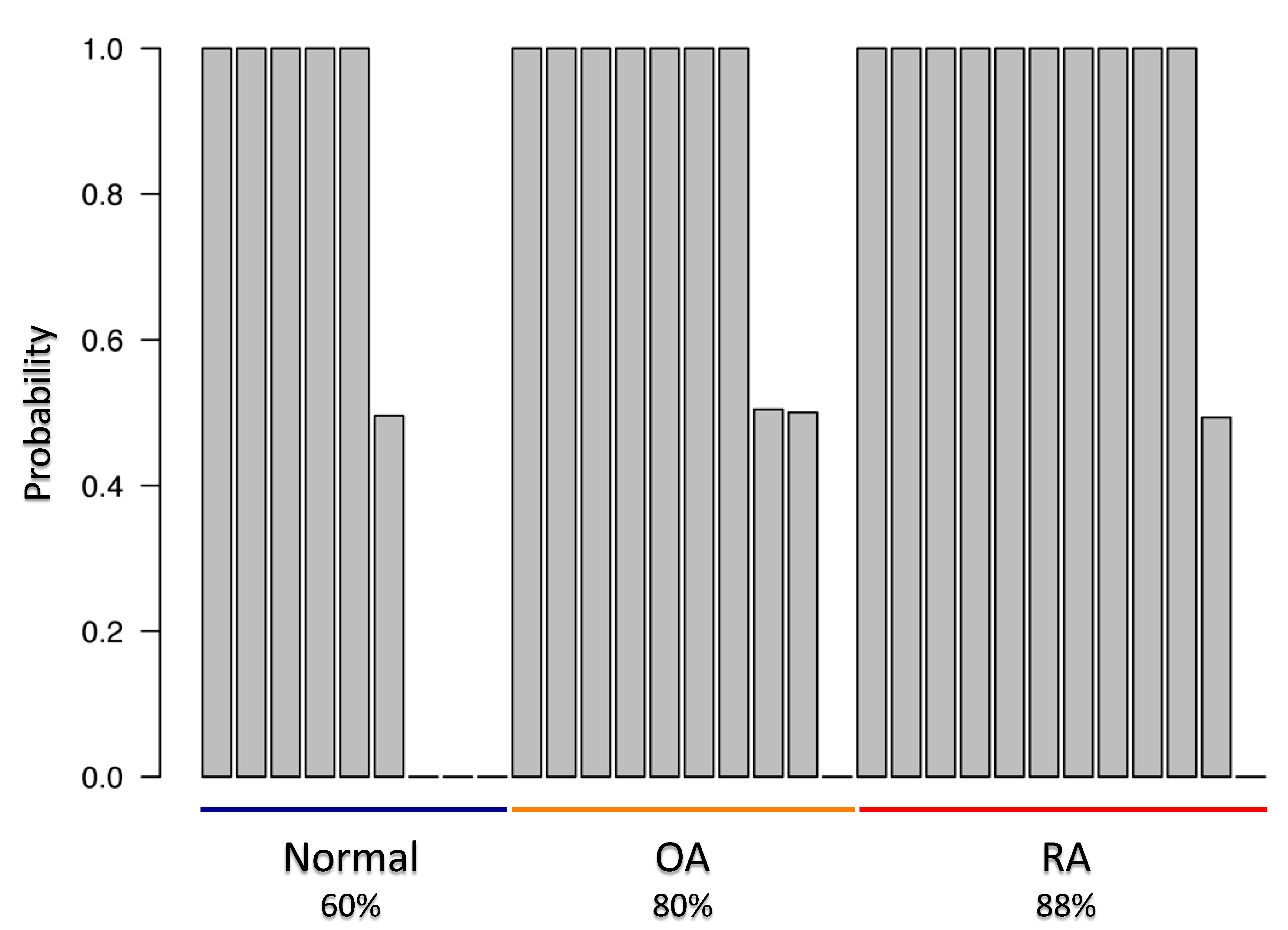

Supplement: S1 Fig — The figure shows the probability of a synovium biopsy sample to be correctly classified as normal, RA or OA on the basis of a canonical serum response signature. The x axis represents each individual sample biopsy in the 3 groups and the y axis the probability of correct classification estimated using a leave one out cross-validation (LOOCV) procedure. The percentage of correct classification using a majority rule is indicated below the sample labels. (TIFF) [file pone.0120917.s001.tiff]

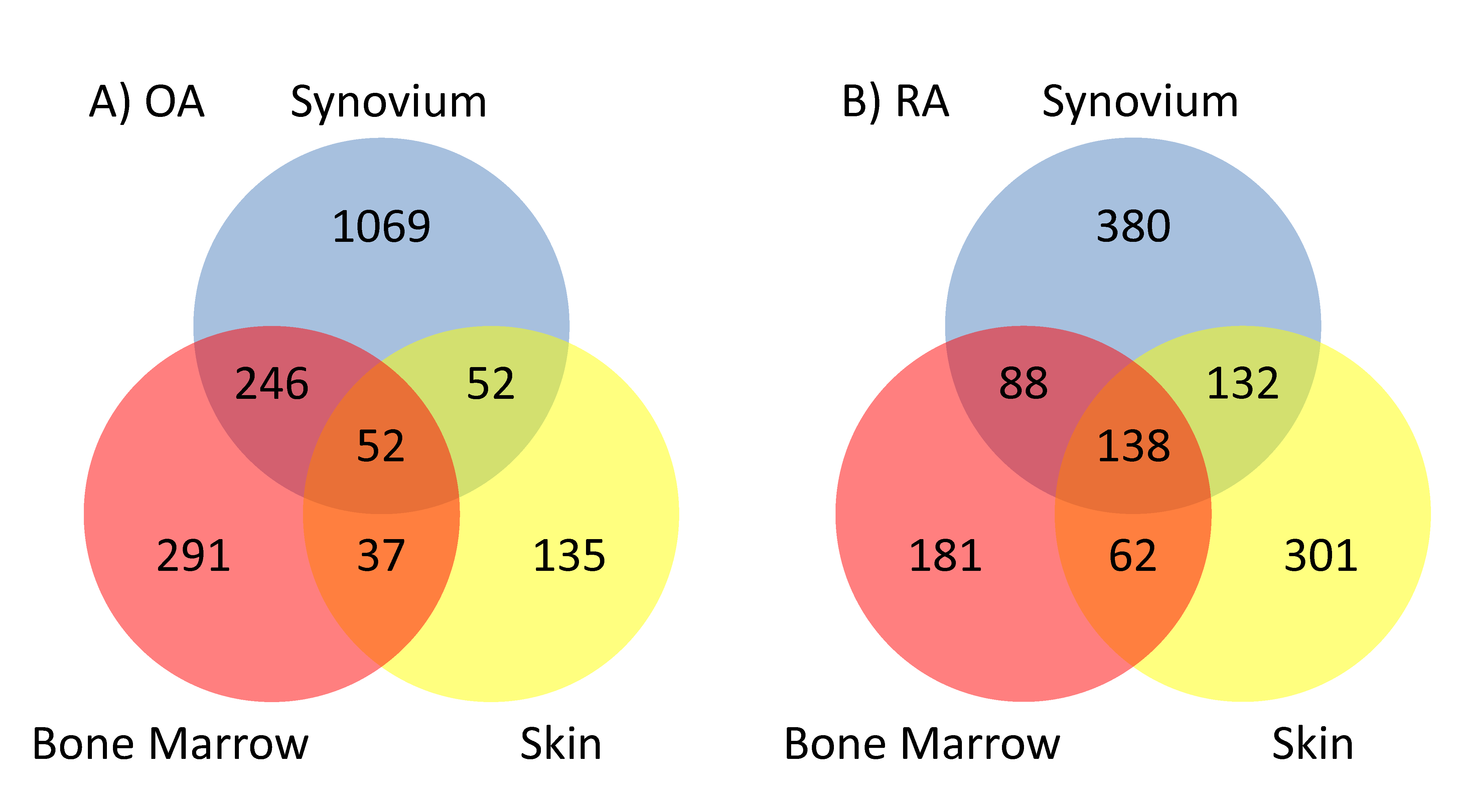

Supplement: S2 Fig — A) Shows the Venn diagram for the response to serum in all tissues in OA. B) represents the serum response of all tissues in RA patients. (TIFF) [file pone.0120917.s002.tiff]
