# Supplementary material for: Stromal Transcriptional Profiles Reveal Hierarchies of Anatomical Site, Serum Response and Disease and Identify Disease Specific Pathways
Source: PLoS One. 2015 Mar 25;10(3):e0120917. doi: 10.1371/journal.pone.0120917 (PMC4373951; doi:10.1371/journal.pone.0120917)
Supplement: S1 Table — RhF, rheumatoid factor; DMARD, Disease-modifying antirheumatic drug; MTX, methotrexate, weekly dose; D-PEN, D-penicillamine; Gold, intramuscular gold, monthly dose; AZA, azathioprine; SSA, sulphasalazine. (DOCX) [file pone.0120917.s003.docx]

**Supplementary Table S1** Patient demographic and disease data

| Patient | Joint | Sex, age | RhF/  erosion | Disease duration (yrs) | DMARD | Prednisolone | DAS28 |
| --- | --- | --- | --- | --- | --- | --- | --- |
| RA patients | | | | | | | |
| RA01 | Knee | F, 62 | +/+ | 15 | MTX |  | 5.5 |
| RA02 | Knee | F, 52 | +/+ | 20 | MTX |  | 4.4 |
| RA03 | Knee | M, 66 | +/+ | 8 | Adalimumab | 5mg | 5.5 |
| RA04 | Knee | F, 71 | +/+ | 16 | D-PEN |  | 5.3 |
| RA05 | Knee | F, 62 | +/+ | 20 | nil |  | 6.8 |
| RA07 | Knee | F, 71 | +/+ | 1 | MTX | 10mg | 7.2 |
| RA08 | Knee | F, 59 | +/+ | 15 | MTX | 15mg | 6.8 |
| RA12 | Hip | F, 32 | -/- | 3 | Gold |  | 4.8 |
| RA13 | Knee | F, 67 | -/+ | 15 | MTX |  | 4.3 |
| RA14 | Knee | F, 62 | +/+ | 23 | Gold | 5mg | 5.5 |
| RA16 | Knee | F, 30 | +/+ | 15 | AZA | 3mg | 6.5 |
| RA17 | Knee | M, 77 | +/+ | 22 | SSA | 5mg | 4.0 |
| OA patients | | | | | | | |
| OA01 | Knee | F, 63 | -/- | 8 |  |  |  |
| OA02 | Knee | F, 67 | -/- | 8 |  |  |  |
| OA03 | Knee | F, 89 | -/- | 10 |  |  |  |
| OA04 | Hip | M, 71 | -/- | 7 |  |  |  |
| OA06 | knee | M. 71 | -/- | 10 |  |  |  |
| OA07 | Knee | F, 73 | -/- | 15 |  |  |  |

RhF, rheumatoid factor; DMARD, Disease-modifying antirheumatic drug; MTX, methotrexate, weekly dose; D-PEN, D-penicillamine; Gold, intramuscular gold, monthly

dose; AZA, azathioprine; SSA, sulphasalazine.
