# Supplementary material for: Stromal Transcriptional Profiles Reveal Hierarchies of Anatomical Site, Serum Response and Disease and Identify Disease Specific Pathways
Source: PLoS One. 2015 Mar 25;10(3):e0120917. doi: 10.1371/journal.pone.0120917 (PMC4373951; doi:10.1371/journal.pone.0120917)
Supplement: S2 Table — Data were obtained from 3 synovial fibroblast lines. qPCR, quantitative PCR; PIAS1, protein inhibitor of activated STAT-1 (signal transducer and activator of transcription-1). (DOCX) [file pone.0120917.s004.docx]

**Supplementary Table S2**: Fold changes in gene expression in OA versus RA synovial fibroblasts in low serum

| Gene | Array fold change | qPCR fold change |
| --- | --- | --- |
| Slit3 | 2.49 | 1.65 |
| PIAS1 | 1.9 | 8.21 |
| Tensin3 | -1.27 | -1.83 |

Data were obtained from 3 synovial fibroblast lines. qPCR, quantitative PCR; PIAS1, protein inhibitor of activated STAT-1 (signal transducer and activator of transcription-1).
