# Supplementary material for: Stromal Transcriptional Profiles Reveal Hierarchies of Anatomical Site, Serum Response and Disease and Identify Disease Specific Pathways
Source: PLoS One. 2015 Mar 25;10(3):e0120917. doi: 10.1371/journal.pone.0120917 (PMC4373951; doi:10.1371/journal.pone.0120917)
Supplement: S3 Table — Overview of the Functional associations in the serum response between RA and OA fibroblasts within each anatomical site. The numbers represent “number of genes increased”/“number of genes decreased” within each term. (DOCX) [file pone.0120917.s005.docx]

Supplementary Table S3: Overview of the Functional associations in the serum response between RA and OA fibroblasts within each anatomical site. The numbers represent “number of genes increased”/”number of genes decreased” within each term.

|  | **Bone Marrow** | | **Skin** | | **Synovium** | |
| --- | --- | --- | --- | --- | --- | --- |
|  | **OA** | **RA** | **OA** | **RA** | **OA** | **RA** |
| **migration of tumor cell lines** | 46/0 | 37/0 | 16/0 | 52/0 | 79/0 | 57/0 |
| **cell movement of tumor cell lines** | 51/0 | 42/0 | 18/0 | 58/0 | 87/0 | 65/0 |
| **cell movement** | 77/0 | 59/0 | 36/0 | 83/0 | 132/0 | 101/0 |
| **migration of cells** | 71/0 | 54/0 | 32/0 | 75/0 | 121/0 | 92/0 |
| **invasion of tumor cell lines** | 47/0 | 32/0 | 19/0 | 50/0 | 74/0 | 61/0 |
| **epithelial-mesenchymal transition of tumor cell lines** | 9/0 | 9/0 | 5/0 | 10/0 | 16/0 | 11/0 |
| **epithelial-mesenchymal transition** | 13/0 | 10/0 | 8/0 | 12/0 | 23/0 | 16/0 |
| **cell survival** | 57/0 | 39/0 | 0/0 | 60/0 | 99/0 | 62/0 |
| **proliferation of cells** | 141/0 | 108/0 | 0/0 | 152/0 | 263/0 | 183/0 |
| **proliferation of tumor cell lines** | 86/0 | 68/0 | 0/0 | 96/0 | 167/0 | 105/0 |
| **invasion of cells** | 54/0 | 38/0 | 0/0 | 52/0 | 82/0 | 67/0 |
| **cell viability** | 53/0 | 37/0 | 0/0 | 58/0 | 95/0 | 59/0 |
| **cell viability of tumor cell lines** | 43/0 | 29/0 | 0/0 | 46/0 | 81/0 | 44/0 |
| **cell movement of carcinoma cell lines** | 11/0 | 9/0 | 0/0 | 16/0 | 20/0 | 15/0 |
| **migration of carcinoma cell lines** | 10/0 | 8/0 | 0/0 | 14/0 | 18/0 | 14/0 |
| **invasion of breast cancer cell lines** | 22/0 | 13/0 | 0/0 | 0/0 | 32/0 | 28/0 |
| **invasion of carcinoma cell lines** | 9/0 | 7/0 | 0/0 | 0/0 | 15/0 | 12/0 |
| **cell movement of lung cancer cell lines** | 10/0 | 6/0 | 0/0 | 0/0 | 15/0 | 11/0 |
| **proliferation of pancreatic cancer cell lines** | 10/0 | 9/0 | 0/0 | 0/0 | 17/0 | 0/0 |
| **interphase** | 29/0 | 25/0 | 0/0 | 0/0 | 64/0 | 0/0 |
| **synthesis of DNA** | 18/0 | 11/0 | 0/0 | 0/0 | 25/0 | 0/0 |
| **migration of lung cancer cell lines** | 8/0 | 0/0 | 0/0 | 10/0 | 14/0 | 10/0 |
| **proliferation of lung cancer cell lines** | 17/0 | 0/0 | 0/0 | 0/0 | 32/0 | 26/0 |
| **alignment of chromosomes** | 0/0 | 6/0 | 0/0 | 8/0 | 10/0 | 11/0 |
| **apoptosis of squamous cell carcinoma cell lines** | 0/0 | 0/6 | 0/0 | 0/0 | 0/10 | 0/8 |
| **M phase of tumor cell lines** | 15/0 | 13/0 | 0/0 | 16/0 | 0/0 | 21/0 |
| **cytokinesis** | 15/0 | 0/0 | 0/0 | 15/0 | 0/0 | 21/0 |
| **invasion of lung cancer cell lines** | 8/0 | 0/0 | 0/0 | 0/0 | 0/0 | 10/0 |
| **apoptosis of cervical cancer cell lines** | 0/0 | 0/0 | 0/0 | 0/19 | 0/0 | 0/30 |
| **chromosomal congression of chromosomes** | 0/0 | 0/0 | 0/0 | 4/0 | 0/0 | 5/0 |
| **cell movement of breast cancer cell lines** | 21/0 | 15/0 | 0/0 | 0/0 | 0/0 | 0/0 |
| **migration of breast cancer cell lines** | 20/0 | 14/0 | 0/0 | 0/0 | 0/0 | 0/0 |
| **colony formation of tumor cell lines** | 18/0 | 12/0 | 0/0 | 0/0 | 0/0 | 0/0 |
| **colony formation** | 28/0 | 18/0 | 0/0 | 0/0 | 0/0 | 0/0 |
| **proliferation of ovarian cancer cell lines** | 10/0 | 8/0 | 0/0 | 0/0 | 0/0 | 0/0 |
| **colony formation of cells** | 26/0 | 16/0 | 0/0 | 0/0 | 0/0 | 0/0 |
| **proliferation of breast cancer cell lines** | 28/0 | 22/0 | 0/0 | 0/0 | 0/0 | 0/0 |
| **DNA replication** | 11/0 | 0/0 | 0/0 | 0/0 | 0/0 | 0/0 |
| **protein kinase cascade** | 20/0 | 0/0 | 0/0 | 0/0 | 0/0 | 0/0 |
| **Viral Infection** | 74/0 | 0/0 | 0/0 | 0/0 | 0/0 | 0/0 |
| **MAPKKK cascade** | 10/0 | 0/0 | 0/0 | 0/0 | 0/0 | 0/0 |
| **G1/S phase transition** | 12/0 | 0/0 | 0/0 | 0/0 | 0/0 | 0/0 |
| **metabolism of DNA** | 18/0 | 0/0 | 0/0 | 0/0 | 0/0 | 0/0 |
| **S phase of tumor cell lines** | 10/0 | 0/0 | 0/0 | 0/0 | 0/0 | 0/0 |
| **colony formation of breast cancer cell lines** | 8/0 | 0/0 | 0/0 | 0/0 | 0/0 | 0/0 |
| **cytokinesis of tumor cell lines** | 10/0 | 0/0 | 0/0 | 0/0 | 0/0 | 0/0 |
| **M phase** | 26/0 | 0/0 | 0/0 | 0/0 | 0/0 | 0/0 |
| **proliferation of carcinoma cells** | 4/0 | 0/0 | 0/0 | 0/0 | 0/0 | 0/0 |
| **development of cardiovascular system** | 0/0 | 25/0 | 0/0 | 0/0 | 0/0 | 0/0 |
| **leukocyte migration** | 0/0 | 16/0 | 0/0 | 0/0 | 0/0 | 0/0 |
| **development of blood vessel** | 0/0 | 24/0 | 0/0 | 0/0 | 0/0 | 0/0 |
| **differentiation of tumor cell lines** | 0/0 | 14/0 | 0/0 | 0/0 | 0/0 | 0/0 |
| **synthesis of eicosanoid** | 0/0 | 9/0 | 0/0 | 0/0 | 0/0 | 0/0 |
| **tubulation of cells** | 0/0 | 7/0 | 0/0 | 0/0 | 0/0 | 0/0 |
| **synthesis of prostaglandin** | 0/0 | 8/0 | 0/0 | 0/0 | 0/0 | 0/0 |
| **transmigration of cells** | 0/0 | 7/0 | 0/0 | 0/0 | 0/0 | 0/0 |
| **mitogenesis** | 0/0 | 7/0 | 0/0 | 0/0 | 0/0 | 0/0 |
| **proliferation of smooth muscle cells** | 0/0 | 11/0 | 0/0 | 0/0 | 0/0 | 0/0 |
| **angiogenesis** | 0/0 | 16/0 | 0/0 | 0/0 | 0/0 | 0/0 |
| **organization of cytoskeleton** | 0/0 | 27/0 | 0/0 | 0/0 | 0/0 | 0/0 |
| **organization of cytoplasm** | 0/0 | 29/0 | 0/0 | 0/0 | 0/0 | 0/0 |
| **synthesis of fatty acid** | 0/0 | 10/0 | 0/0 | 0/0 | 0/0 | 0/0 |
| **neoplasia of cells** | 0/0 | 7/0 | 0/0 | 0/0 | 0/0 | 0/0 |
| **synthesis of prostaglandin E2** | 0/0 | 7/0 | 0/0 | 0/0 | 0/0 | 0/0 |
| **growth of epithelial tissue** | 0/0 | 21/0 | 0/0 | 0/0 | 0/0 | 0/0 |
| **vasculogenesis** | 0/0 | 23/0 | 0/0 | 0/0 | 0/0 | 0/0 |
| **synthesis of lipid** | 0/0 | 20/0 | 0/0 | 0/0 | 0/0 | 0/0 |
| **fatty acid metabolism** | 0/0 | 13/0 | 0/0 | 0/0 | 0/0 | 0/0 |
| **neoplasia of tumor cell lines** | 0/0 | 6/0 | 0/0 | 0/0 | 0/0 | 0/0 |
| **migration of leukemia cell lines** | 0/0 | 5/0 | 0/0 | 0/0 | 0/0 | 0/0 |
| **cell movement of myeloid cells** | 0/0 | 11/0 | 0/0 | 0/0 | 0/0 | 0/0 |
| **invasion of squamous cell carcinoma cell lines** | 0/0 | 5/0 | 0/0 | 0/0 | 0/0 | 0/0 |
| **cell movement of squamous cell carcinoma cell lines** | 0/0 | 5/0 | 0/0 | 0/0 | 0/0 | 0/0 |
| **activation of cells** | 0/0 | 18/0 | 0/0 | 0/0 | 0/0 | 0/0 |
| **chemotaxis of myeloid cells** | 0/0 | 10/0 | 0/0 | 0/0 | 0/0 | 0/0 |
| **chemotaxis of cells** | 0/0 | 18/0 | 0/0 | 0/0 | 0/0 | 0/0 |
| **invasion of breast cell lines** | 0/0 | 4/0 | 0/0 | 0/0 | 0/0 | 0/0 |
| **apoptosis of tumor cell lines** | 0/0 | 0/49 | 0/0 | 0/0 | 0/0 | 0/0 |
| **cell death of tumor cell lines** | 0/0 | 0/55 | 0/0 | 0/0 | 0/0 | 0/0 |
| **cell cycle progression** | 0/0 | 40/0 | 0/0 | 0/0 | 103/0 | 0/0 |
| **cell viability of lung cancer cell lines** | 0/0 | 0/0 | 0/0 | 0/0 | 0/0 | 11/0 |
| **proliferation of fibroblasts** | 0/0 | 0/0 | 0/0 | 0/0 | 0/0 | 14/0 |
| **adhesion of tumor cell lines** | 0/0 | 0/0 | 0/0 | 0/0 | 0/0 | 26/0 |
| **proliferation of carcinoma cell lines** | 0/0 | 0/0 | 0/0 | 0/0 | 0/0 | 24/0 |
| **proliferation of connective tissue cells** | 0/0 | 0/0 | 0/0 | 0/0 | 0/0 | 25/0 |
| **adhesion of breast cancer cell lines** | 0/0 | 0/0 | 0/0 | 0/0 | 0/0 | 9/0 |
| **cell movement of endothelial cells** | 0/0 | 0/0 | 0/0 | 0/0 | 0/0 | 28/0 |
| **proliferation of dermal cells** | 0/0 | 0/0 | 0/0 | 0/0 | 0/0 | 8/0 |
| **interphase of cervical cancer cell lines** | 0/0 | 0/0 | 0/0 | 0/0 | 0/0 | 9/0 |
| **necrosis** | 0/0 | 0/0 | 0/0 | 0/0 | 0/0 | 0/128 |
| **cell death of carcinoma cell lines** | 0/0 | 0/0 | 0/0 | 0/0 | 0/0 | 0/17 |
| **growth of connective tissue** | 0/0 | 14/0 | 0/0 | 0/0 | 0/0 | 26/0 |
| **proliferation of epithelial cells** | 0/0 | 8/0 | 0/0 | 0/0 | 0/0 | 13/0 |
| **migration of endothelial cells** | 0/0 | 0/0 | 8/0 | 0/0 | 0/0 | 26/0 |
| **interphase of tumor cell lines** | 0/0 | 0/0 | 0/0 | 0/0 | 42/0 | 0/0 |
| **cell cycle progression of tumor cell lines** | 0/0 | 0/0 | 0/0 | 0/0 | 30/0 | 0/0 |
| **sensitization of cells** | 0/0 | 0/0 | 0/0 | 0/0 | 7/0 | 0/0 |
| **development of body trunk** | 0/0 | 0/0 | 0/0 | 0/0 | 19/0 | 0/0 |
| **invasion of pancreatic cancer cell lines** | 0/0 | 0/0 | 0/0 | 0/0 | 9/0 | 0/0 |
| **growth of plasma membrane projections** | 0/0 | 0/0 | 0/0 | 0/0 | 12/0 | 0/0 |
| **cell death** | 0/0 | 0/0 | 0/0 | 0/0 | 0/235 | 0/0 |
| **cell death of squamous cell carcinoma cell lines** | 0/0 | 0/0 | 0/0 | 0/0 | 0/11 | 0/0 |
| **S phase** | 16/0 | 0/0 | 0/0 | 0/0 | 27/0 | 0/0 |
| **cell movement of pancreatic cancer cell lines** | 0/0 | 0/0 | 0/0 | 0/0 | 8/0 | 7/0 |
| **cell viability of carcinoma cell lines** | 0/0 | 0/0 | 0/0 | 0/0 | 15/0 | 12/0 |
| **formation of mitotic spindle** | 0/0 | 0/0 | 0/0 | 0/0 | 0/9 | 0/9 |
| **migration of brain cancer cell lines** | 0/0 | 0/0 | 0/0 | 10/0 | 0/0 | 0/0 |
| **cell movement of brain cancer cell lines** | 0/0 | 0/0 | 0/0 | 11/0 | 0/0 | 0/0 |
| **homing of tumor cell lines** | 0/0 | 0/0 | 0/0 | 10/0 | 0/0 | 0/0 |
| **adhesion of epithelial cells** | 0/0 | 0/0 | 0/0 | 9/0 | 0/0 | 0/0 |
| **chemotaxis of tumor cell lines** | 0/0 | 0/0 | 0/0 | 9/0 | 0/0 | 0/0 |
| **proliferation of hepatoma cell lines** | 0/0 | 0/0 | 0/0 | 16/0 | 0/0 | 0/0 |
| **adhesion of kidney cells** | 0/0 | 0/0 | 0/0 | 7/0 | 0/0 | 0/0 |
| **cell death of kidney cells** | 0/0 | 0/0 | 0/0 | 19/0 | 0/0 | 0/0 |
| **shape change of embryonic cell lines** | 0/0 | 0/0 | 0/0 | 4/0 | 0/0 | 0/0 |
| **activation of vascular endothelial cells** | 0/0 | 4/0 | 4/0 | 5/0 | 0/0 | 0/0 |
| **cell movement of leukemia cell lines** | 0/0 | 10/0 | 0/0 | 10/0 | 0/0 | 0/0 |
